# Conformational Dynamics of Ago-Mediated Silencing Processes

**DOI:** 10.3390/ijms160714769

**Published:** 2015-07-01

**Authors:** Sarah Willkomm, Tobias Restle

**Affiliations:** Institute of Molecular Medicine, Universitätsklinikum Schleswig-Holstein, Universität zu Lübeck, Lübeck 23538, Germany; E-Mail: willkomm@imm.uni-luebeck.de

**Keywords:** Argonaute, mechanism, RNA interference, dynamics, kinetics, pre-steady state, steady state

## Abstract

Argonaute (Ago) proteins are key players of nucleic acid-based interference mechanisms. Their domains and structural organization are widely conserved in all three domains of life. However, different Ago proteins display various substrate preferences. While some Ago proteins are able to use several substrates, others are limited to a single one. Thereby, they were demonstrated to act specifically on their preferred substrates. Here, we discuss mechanisms of Ago-mediated silencing in relation to structural and biochemical insights. The combination of biochemical and structural information enables detailed analyses of the complex dynamic interplay between Ago proteins and their substrates. Especially, transient binding data allow precise investigations of structural transitions taking place upon Ago-mediated guide and target binding.

## 1. Introduction

Argonaute (Ago) proteins are found in all three domains of life [1]. Even though they were initially discovered in eukaryotes [2], first structural insights stem from their prokaryotic counterparts [3,4,5,6,7]. In eukaryotes, Ago proteins are the key players of a process for posttranscriptional regulation of gene expression termed RNA interference (RNAi) [8,9]. This process involves short double-stranded RNAs which can be divided into two main subgroups: small interfering RNAs (siRNAs) and microRNAs (miRNAs). In contrast to siRNAs, miRNAs are partially complementary duplexes which merely need seed complementarity to their target mRNA to silence gene expression [10,11]. Ago-mediated target mRNA cleavage, in eukaryotes induced by siRNAs, requires base pairing beyond the seed [12,13]. In humans, only hArgonaute2 (hAgo2) is able to cleave target RNAs [14,15]. Both classes of small RNAs are processed from double-stranded long precursor RNAs by the RNase III-like endonuclease Dicer [16,17]. The mature siRNAs or miRNAs are loaded into Ago within a multiprotein complex termed RNA-induced silencing complex (RISC)-loading complex (RLC) consisting of Dicer and a dsRNA-binding protein which can be either TAR RNA binding protein (TRBP) or protein activator of PKR (PACT) [18,19,20,21,22]. In the next step, one of the strands of the short double-stranded RNA, called passenger strand, is removed. Depending on the degree of complementarity of the miRNA or rather siRNA, the passenger strand is cleaved before unwinding [23,24,25]. The other strand, called guide RNA, is retained in the Ago protein in a complex termed RISC [26]. Guided by this single-stranded RNA, Ago binds a matching target mRNA. In case of siRNAs, the target mRNA is subject to Ago-mediated cleavage [27], whereas miRNAs mainly lead to interference with the translational machinery [28,29,30,31]. In opposition to eukaryotic Agos, their prokaryotic counterparts often use DNA as guide as well as target substrates [4,6,32,33,34,35]. Supposedly, they are involved in the defense against invading foreign genetic elements [35], but in contrast to eukaryotic Ago proteins, most mechanistic and functional details remain to be elucidated [1,36].

This review discusses the mechanisms of Ago-mediated silencing related to biochemical and structural insights. A series of *T. thermophilus* Ago (TtAgo) X-ray crystal structures [5,6,7,13] as well as recent structures of eukaryotic Ago proteins [37,38,39,40,41] shed light on the complex processes that occur during Ago-mediated binding of guide and target RNAs and subsequent cleavage of target strands. In combination with biochemical data, this structural information enables insights into the dynamics of Ago-mediated silencing.

## 2. Functional Loading of Ago Proteins with Guide Strands

### 2.1. The Guide 5′-End Mainly Determines the Affinity of Binary Complexes

**Figure 1:**
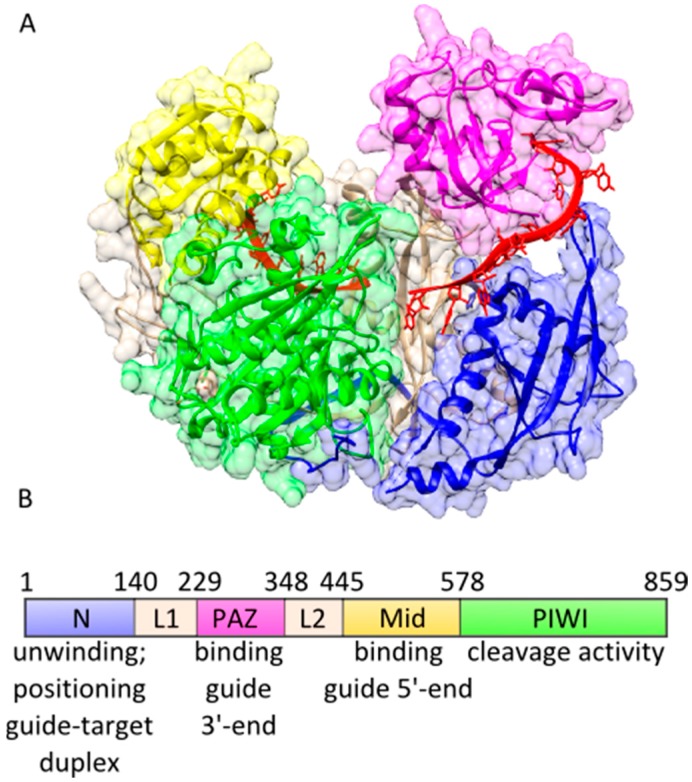
Domain organization of hAgo2. (**A**) X-ray structure of a binary hAgo2-guide complex (pdb: 4W5N) with the domains coloured individually. The guide strand is depicted in red; and (**B**) Schematic representation of the hAgo2 domains with their individual functions assigned.

The 5′-nucleotide of guide strands was shown to be critical for the association of Ago proteins with guide strands [33,40,42,43,44,45]. In-depth pre-steady state studies of the formation of binary hAgo2-guide complexes indicate that binding of the guide 5′-end is a prerequisite for the correct positioning of the guide in the nucleic acid binding channel of hAgo2 [46]. Furthermore, these transient binding data indicate that in comparison to the N-lobe consisting of the PAZ and the N-terminal domain, the C-lobe comprising the Mid and the PIWI domain is easily accessible for the incoming guide strand, since binding to the Mid domain is significantly faster than the subsequent positioning of the 3′-portion of the guide strand, followed by PAZ association of the guide 3′-end (Table 1). This is corroborated by structural information from hAgo2 showing that the guide 3′-portion is threaded through a very narrow channel in the N-lobe of hAgo2 [39] (Figure 1).

**Table 1:** Summary of rate constants measured for formation of binary hAgo2-guide, AaAgo-guide and hAgo2-PAZ9-guide complexes. Dissociation constants calculated from association and dissociation constants are displayed. Cartoons are based on hAgo2 X-ray structures with individual domains coloured. The guide substrate is depicted in blue with the 5′-end indicated by the phosphate group. Relative positions of the guide substrate are indicated. n.d. = values not determined in this study.

Collision Complex				5′-End Binding			3′-End Binding	
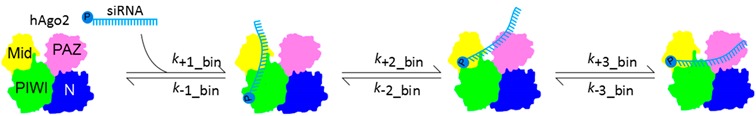								
**Ago Protein**	***k*_1_bin_** **(M^−1^·s^−1^)**	***k*_-1_bin_** **(s^−1^)**	***k*** **_2_bin_** **(s^−1^)**	***k*_-2_bin_** **(s^−1^)**	***k*_3_bin_** **(s^−1^)**	***k*_-3_bin_** **(s^−1^)**	***K*_D_bin_** **(nM)**	**Reference**
**wt hAgo2**	0.6 × 10^8^	6.2	0.26	0.17	0.012	0.007	37	[46]
**wt hAgo2**	1.2 × 10^5^	n.d.	n.d.	n.d.	n.d.	0.007	57	[47]
**AaAgo**	n.d.	n.d.	n.d.	n.d.	n.d.	0.004	10	[4,48]
**hAgo2-PAZ9**	0.2 × 10^8^	7.8	0.18	0.024	–	–	49.5	[46]

**Figure 2:**
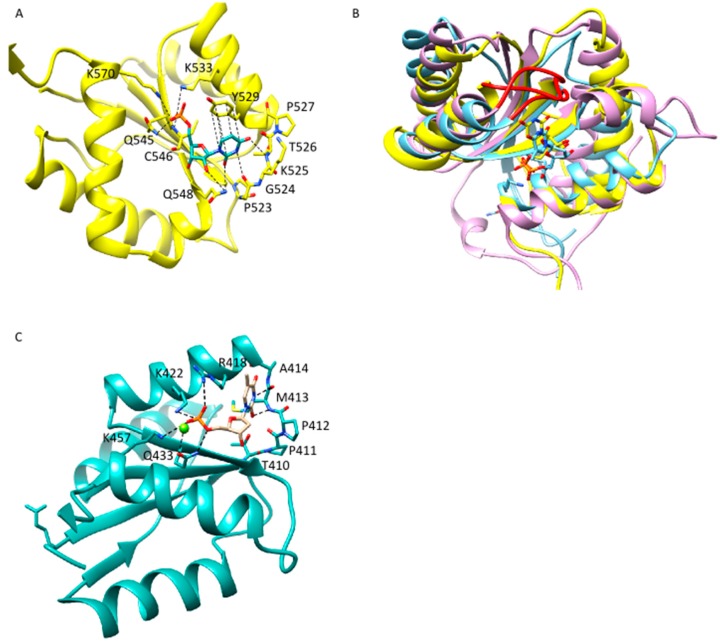
Binding of the guide 5′-end by the Mid domain of Ago. (**A**) Mid domain of hAgo2 (pdb: 4W5N) in complex with a 5′-uracil of the guide RNA. Residues interacting with the 5′-phosphate and the 5′-base are highlighted according to Frank *et al.* [42]. Backbone of the nucleotide specificity loop (NSL) is depicted in atoms/bonds representation; (**B**) Overlay of the Mid domains of hAgo2 (pdb: 4W5N) in yellow, TtAgo (pdb: 3DLH) in blue and *P. furiosus* Ago (PfAgo) (pdb: 1U04) in purple. The regions corresponding to the NSL are highlighted in red; (**C**) Mid domain of TtAgo in complex with a 5′-thymin. Residues possibly interacting with the 5′-nucleotide are highlighted according to Frank *et al.* [42]. Backbone of the region corresponding to the NSL is depicted in atom/bond representation.

The 5′-phosphate undergoes several tight and specific interactions with residues of the Mid (Figure 2A–C) and the PIWI domain [5,33,38,40,42,44] which make it an important determinant for the positioning of a guide strand within Ago proteins [40,45]. The interactions with the 5′-phosphate are stronger and more specific than any other interaction between the Mid binding pocket and the guide 5′-end [40]. This explains significantly decreased affinities of Ago proteins for guide strands of a factor of 5–15 if the 5′-terminal phosphate is missing (Table 2). Even a double-stranded siRNA, which requires larger structural transitions of the Ago protein to allow positioning within the nucleic acid binding channel of Ago, is bound more tightly by hAgo2 than the unphosphorylated single-stranded guide (Table 2). The mutation of only two of the 5′-phosphate-interacting residues in the Mid binding pocket of hAgo2 leads to severely impaired cleavage efficiency of hAgo2, underscoring the importance of the residues in the Mid binding pocket [5,33]. Interestingly, this does not seem to be exclusively attributed to a destabilization of the 5′-phosphate in the Mid domain. Despite reduced cleavage efficiency, a guide strand without a 5′-phosphate is as well as its phosphorylated counterpart able to guide hAgo2-mediated cleavage of target RNAs [45]. This indicates a dynamic adaption of the Mid binding pocket during binding of the guide 5′-end which is dependent on the presence of the phosphate-interacting Mid domain residues.

In addition to interactions with the 5′-phosphate of the guide strand, interactions with the terminal base are described. Different Ago proteins display preferences for certain 5′-nucleotides [34,49,50,51,52]. Moreover, there are findings that with a certain guide sequence, cleavage of target RNAs mediated by hAgo2 is only possible in presence of a 5′-terminal uracil [53]. This 5′-nucleotide bias was explained by Frank *et al.* [42]. They identified the so-called nucleotide specificity loop (NSL) (Figure 2). A 5′-uracil forms stabilizing interactions with the backbone of this loop, whereas interactions with a 5′-adenine are weaker to the point of repulsive interactions in the presence of a 5′-cytosine or 5′-guanine (Table 2). However, in compliance with the conclusion of Elkayam *et al.* [40] that there are no interactions with the guide 5′-end that are comparable to the ones with the terminal phosphate, binding affinity of hAgo2 for guides with an abasic 5′-end is only reduced by a factor of 2 (Table 2). This indicates the discrimination between different 5′-nucleotides by the NSL of hAgo2 might not be as stringent as expected from the affinities of the isolated recombinantly expressed hAgo2 Mid binding pocket for the four possible different nucleotide monophosphates (Table 2). Whereas binding experiments conducted with the isolated hAgo2 Mid domain display a clear bias for a 5′-uracil or 5′-adenine (Table 2), own biochemical data from experiments with the entire hAgo2 protein and full-length guide strands indicate that the Mid binding pocket is able to adapt to all four different 5′-nucleotides (unpublished data). These data underpin the idea of a dynamic adaptation of the interaction network between different 5′-nucleotides and the Mid binding pocket. Further support comes from discoveries made with TtAgo. While structural data show that the region corresponding to the NSL is organized in a similar fashion compared to hAgo2 [42] (Figure 2) and stabilizing interactions between a 5′-thymine and this loop can be detected [42], Swarts *et al.* [34] provided experimental evidence that there is a bias for a terminal cytosine. Moreover, they demonstrated TtAgo cleavage efficiency is equally guided by guide strands with all four possible 5′-nucleotides. Swarts *et al.* [34] postulate that in case of TtAgo the reason for the 5′-cytosine bias could be a special selection mechanism by TtAgo as well as an upstream processing mechanism resulting in preferential production of small RNAs carrying a 5′-cytosine.

Since the thermodynamically more unstable end is selected as the 5′-end of a miRNA [54,55], the less stable interaction between a UA in comparison to a GC base pair could contribute to the selection mechanism of hAgo2. Additionally, a binding pocket for the nucleotide opposite of the guide 5′-end specifically recognizes adenine [39]. For target strands, this leads to three-fold higher affinity if an adenine is present in this position. Also for siRNAs, this pocket might contribute to an improved affinity if the passenger is carrying an adenine in this critical position. In *A. thaliana* where Ago proteins have to select the correct RNA out of a subset of diverse small RNA classes, the NSL seems to be more important. Interactions between the 5′-terminal base with the NSL of *A. thaliana* Ago (AtAgo) are also involving special residues of the NSL which are helping to discriminate between different 5′-bases [43]. In contrast, in archaeal Ago proteins the NSL is probably positioned too far away from the 5′-terminal nucleotide to interact with the 5′-terminal base [42].

**Table 2:** Binding affinities of different Ago proteins for various guide substrates.

Guide Substrate	*K*_D_ (nM)	Reference
**hAgo2**		
19-Mer guide RNA	83	[47]
21-Mer guide RNA	7	[46]
OH-19-Mer guide RNA	395	[47]
OH-21-Mer guide RNA	106	[46]
19-Mer guide RNA Pos 1 abasic	225	[47]
Blunt end 19-Mer dsRNA	6297	[47]
21-Mer siRNA	48	[46]
19-Mer DNA	565	[47]
**Methoxyethyl-Substituted Guides**		
19-Mer pos 1–3	1100	[47]
19-Mer pos 12–14	234	[47]
**hAgo2 Mid Domain**		
UMP	1.2 × 10^5^	[42]
AMP	2.6 × 10^5^	[42]
CMP	3.6 × 10^6^	[42]
GMP	3.3 × 10^6^	[42]
**DmAgo1**		
23-Mer guide RNA	2.9	[56]
**DmAgo1-PAZ6**		
23-Mer guide RNA	2.3	[56]
**DmAgo1-∆N-PAZ**		
23-Mer guide RNA	2.7	[56]
**DmAgo2**		
23-Mer guide RNA	10	[56]
**DmAgo2-∆N-PAZ**		
23-Mer guide RNA	9.5	[56]
**AaAgo**		
ssDNA	10	[4]
ssDNA	2.9–270	[48]
dsDNA	1000	[4]
ssRNA	970	[4]
dsRNA	>10,000	[4]
DNA/RNA	640	[4]

Besides very specific and tight interactions with the 5′-end of the guide strand, the entire 5′-portion of the guide strand interacts with Ago, significantly contributing to binding affinity as judged from the difference in dissociation constants of NMPs in comparison to full-length guide strands (Table 2). Since guide strands are bound by Ago in a sequence-independent manner, most interactions can be detected with the backbone of the guide strand [37,38,40]. There are interactions between Ago residues and every phosphate of the seed backbone and two adjacent nucleotides as well as RNA-specific interactions with the 2′-hydroxyl of the sugar moiety leading to an A form-like conformation of the guide’s seed-region. Missing interactions with the 2′-hydroxyl in case of DNA guides could explain the significantly increased dissociation constant of binary hAgo2-DNA guide complexes in contrast to hAgo2-RNA guide complexes (Table 2).

### 2.2. Anchoring of the Guide 3′-End Is Decisive for the Formation of Functional Binary Ago-Guide Complexes

**Figure 3:**
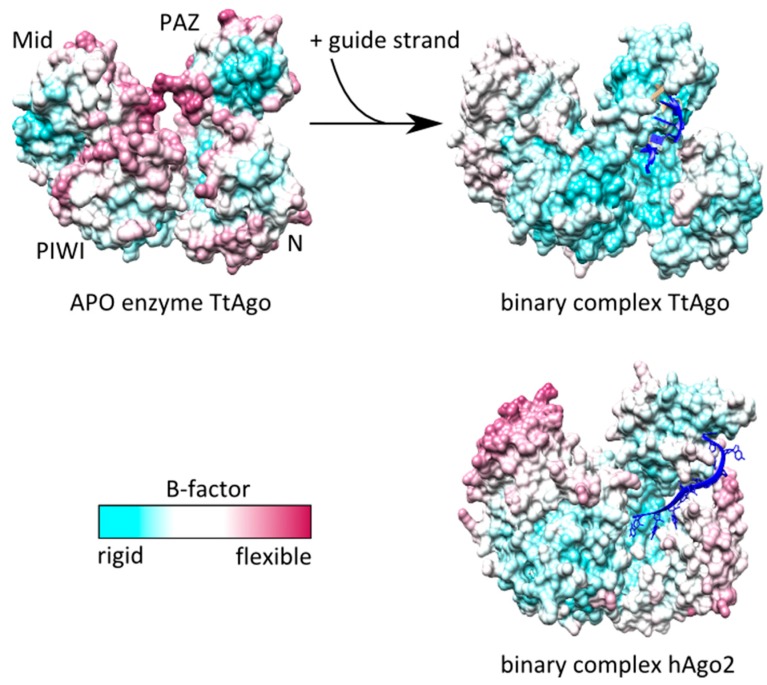
B-factor colouring of a TtAgo APO enzyme, binary TtAgo-guide and hAgo2-guide complexes. B-factor colouring was conducted using Chimera 1.7. APO-enzyme (pdb: 3DLB), binary complex TtAgo (pdb: 3DLH) and hAgo2 (pdb: 4W5N).

Crystal structures of prokaryotic and eukaryotic Ago proteins reveal that in binary Ago-guide complexes the 3′-end of the guide is fixed to the Ago protein [7,37,38,40]. It is anchored in the PAZ domain, where it stays attached until binding to a complementary target strand [6,13,32,57]. Molecular Dynamics (MD) simulations demonstrated that the *A. aeolicus* Ago (AaAgo) N and PAZ domain undergo concerted periodic motions in the unbound state whereas Mid and PIWI remain relatively stable [48]. These domain motions are important to open the protein for an incoming nucleic acid [58]. Binding of a guide strand is governing the Ago protein into a stable conformation. This process of stabilization during binding of the guide strand is underscored by B-factors taken from TtAgo crystal structures (Figure 3). Whereas in the unbound state there are large areas displaying a high B-factor indicating flexibility, the binding to a guide strand leads to a freezing of Ago motion especially in the N-lobe of Ago. Although prokaryotic and eukaryotic Ago proteins are highly homologous concerning domain organization and structure [40], there are some differences between binary complexes of prokaryotic or rather eukaryotic Ago proteins. Whereas in case of TtAgo the B-factors of the entire protein are very low with the exception of the Mid domain, in hAgo2 only PIWI and PAZ domain have low B-factors while the Mid as well as the N-terminal domain display high B-factors indicating a high degree of flexibility.

Steady state binding measurements demonstrate that the guide 3′-end only marginally contributes to the binding affinity of binary hAgo2-guide complexes [48]. This is corroborated by experiments with the hAgo2-PAZ9 mutant. This protein contains nine point mutations, precluding binding of the guide 3′-end to the PAZ domain [59]. Pre-steady state measurements demonstrate that association of a guide to the Mid domain remains unaffected whereas PAZ binding is abolished [46] (Table 1). However, without PAZ binding the affinity of binary complexes is only minimally reduced [46,60] (Table 1). In *D. melanogaster*, even deletion of the PAZ and additionally the N-terminal domain does not have an effect on binding affinity of binary DmAgo-guide complexes (Table 2).

Transient binding experiments show that guide 3′-end binding to the PAZ domain is one order of magnitude slower compared to the guide 5′-end association with the Mid domain [46] (Table 1). This is consistent with structural data from TtAgo showing that binding of the complete guide strand requires large conformational changes of Ago. Upon guide binding, the Mid and the PAZ domain are rotating by 22° and 25°, respectively [7]. These movements extend the nucleic acid binding channel by 8 Å allowing binding of the complete guide strand. However, in these structures, guide nucleotides 12–17 are not traceable leading to a lack of information about how the 3′-portion of the guide finds its way through the nucleic acid binding channel. Crystallized binary complexes comprising hAgo2 and a guide RNA show that the 3′-portion of the guide strand is positioned in a very narrow channel between the N- and the PAZ domain with the bases facing the interior of the channel [39], which possibly also is a cause for slow PAZ association of the guide 3′-portion.

The crystal structures provide snapshots of the binary complex with both ends of the guide already fixed to the Mid and the PAZ domain but do not inform the process of binding. Pre-steady state binding experiments strongly suggest the guide is binding to the Mid and PAZ domain in a consecutive fashion [46]. Also, dissociation seems to follow this pathway but in the opposite direction. This is corroborated by the fact that dissociation from the Mid domain is significantly slowed down in binary complexes of hAgo2-PAZ9 and a guide RNA. In these complexes, the guide 3′-end is not anchored in the PAZ domain, indicating that dissociation from the PAZ domain is a pre-requisite to trigger conformational changes for guide release from binary complexes.

Although it turns out association between the guide 3′-end and the PAZ domain is not necessary to achieve tight binding of Ago to a guide strand, anchoring of the guide 3′-end in the PAZ domain is an important precondition to form catalytically active ternary Ago-guide-target complexes. This is confirmed by studies conducted with hAgo2-PAZ9 that due to its inability to bind the guide 3′-end with the PAZ domain is not able to perform RNA silencing [61] implying that PAZ association is a prerequisite to position the guide strand in the nucleic acid binding channel. The lack of interaction between the guide 3′-end and the PAZ domain leads to a significantly increased steady state dissociation constant of ternary hAgo2-guide-target complexes (Table 3). Hutvagner *et al.* [9] suggested the PAZ domain could be part of a mechanism to distinguish between fragments of degraded RNA and mature si- or miRNAs.

## 3. Binding of Target Strands to Binary Ago-Guide Complexes

### 3.1. Dynamic Behavior of Ago Is the Basis of Target Turnover

Silencing mediated by Ago proteins requires the assembly of catalytically active ternary complexes comprising Ago, a guide and a target strand. Contacting single-stranded regions in a target strand, Ago identifies accessible potential target sites [62]. Identification of possible target sites might also be enhanced by a target nucleotide binding pocket of Ago binding the target nucleotide opposite the guide 5′-nucleotide [13,39]. In hAgo2, this pocket displays a clear bias for adenines [39]. Another important feature for target recognition is the seed region. The seed region of the Ago-bound guide is prearranged to facilitate base pairing with a matching target strand [7,38,40,63,64]. In contrast, the nucleotides in the 3′-portion are positioned in a narrow channel in the N-lobe of Ago with the base edges not being able to contact a target strand [39]. This situation is reflected by pre-steady state rate constants measured for the binding of target RNA to binary hAgo2-guide RNA complexes [46]. Binding of the target in the seed region is significantly faster than the subsequent extended base pairing in the 3′-region of the guide (Table 3). To allow base pairing in the 3′-region of the guide strand, several structural transitions have to occur [5,6,13,39]. These transitions finally lead to the release of the guide 3′-end from the PAZ domain as observed in bacterial, archaeal and eukaryotic Ago proteins [6,32,46,57]. Transient binding experiments using hAgo2, guide RNA and target RNA revealed the release from the PAZ domain being the rate-limiting step for the formation of catalytically active ternary complexes [46]. In eukaryotic and archaeal Agos, these transitions involve the movement of the so-called helix-7 which otherwise blocks the target-guide interactions beyond guide nucleotide 5 counted from the 5′-end [39] and inserts a kink in the guide strand to interrupt base stacking beyond guide nucleotide 6 by inserting an isoleucine between guide nucleotide 6 and 7 [38,40]. The kink is also present in bacterial Ago proteins, indicating that the presentation of only a few nucleotides of the seed sequence is a universal feature of Ago-mediated target recognition [39]. In hAgo2 ternary complexes, helix-7 contacts the minor groove of the guide-target duplex in the seed region and therefore stabilizes the nucleic acids in hAgo2. A comparable helix is not present in TtAgo, indicating a functional difference between the two proteins [39]. These differences do not necessarily have to be dependent on the substrates (hAgo2 is using RNA guide and target, whereas TtAgo mostly uses DNA [6,34]) since archaeal Ago proteins, which like hAgo2 harbor helix-7, show preferences for DNA guide and target substrates [32].

Most likely the PAZ domain moves together with helix-7 to allow a widening of the N-terminal nucleic acid binding channel and to release the guide 3′-end from the PAZ domain [39]. In addition to a movement of the PAZ domain, parts of the N-terminal domain are involved in correct positioning of the guide strand in the nucleic acid binding channel. Two motifs in the N-terminal domain, which can be either activating or inhibiting, govern the correct positioning of the nucleic acid towards the catalytic tetrad and thereby modulate Ago slicing activity [65]. Information from X-ray structures of hAgo2 in complex with a 21 nt guide RNA and a 11 nucleotide target RNA indicate the N-domain might be involved in the regulation of the position of the magnesium ion which is important for cleavage [39].

Interestingly, a rebinding of the guide 3′-end to the PAZ domain is decisive for effective multiple turnover of Ago. Jung *et al.* [57] recognized that a prevention of guide 3′-binding to the PAZ domain binding pocket disturbs the dissociation rate constant of target strands from ternary TtAgo-guide-target complexes. This implies structural transitions leading to the unwinding of guide and target are taking place upon this “PAZ cycling”. These structural transitions are limiting the rate of target as well as product release [46] as suggested earlier [27,45]. Besides the PAZ domain, the N-terminal domain of Ago plays an important role for the unwinding of the guide-target duplex [65,66,67,68]. As expected, due to high structural homology of Ago proteins among different organisms, the pre-steady state rate constant for this phase is very similar for different Ago proteins (Table 3).

**Table 3:** Summary of rate constants measured for formation of ternary Ago-guide-target complexes. The cartoons are based on hAgo2 X-ray structures with the four domains coloured separately. Guide substrate is depicted in blue with the 5′-end indicated by the phosphate group, whereas the target substrate is depicted in red. Relative positions of guide and target substrates are indicated. n.d. = rate constants not determined in the individual study. If guide-target substrates are not completely complementary, information about complementarity between guide and target strand is given by the remarks “seed” (guide nucleotides 2–8) and “3′-sup” (guide nucleotides 13–16).

		Collision Complex			Seed Pairing					PAZ Release
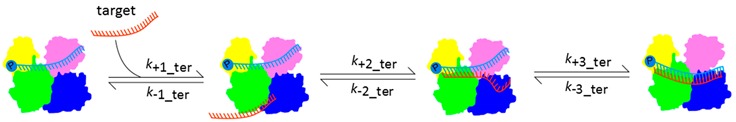										
**Ago Protein and Guide-Target Complementarity**	***k*_1_ter_** **(M^−1^·s^−1^)**		***k*_-1_ter_** **(s^−1^)**	***k*_2_ter_** **(s^−1^)**		***k*_-2_ter_** **(s^−1^)**	***k*_3_ter_** **(s^−1^)**	***k*_-3_ter_** **(s^−1^)**	***K*_D_ter_** **(nM)**	**Reference**
**hAgo2**	3.2 × 10^8^		2.0	0.01		0.002	0.003	0.0002	0.2	[46]
**hAgo2-PAZ9**	2.9 × 10^8^		9.4	0.01		0.02	–	–	47.2	[46]
**Fly Ago2**	0.2 × 10^8^		n.d.	n.d.		n.d.	n.d.	0.00009	0.004	[69]
**Fly Ago2-seed**	2.1 × 10^8^		n.d.	n.d.		0.0045	–	–	210	[69]
**Fly Ago2-seed + 3′-sup**	3.1 × 10^8^		n.d.	n.d.		0.005	–	–	120	[69]
**Mouse Ago2**	0.4 × 10^8^		n.d.	n.d.		n.d.	n.d.	0.0008	0.02	[69]
**Mouse Ago2-seed**	0.2 × 10^8^		n.d.	n.d.		0.0005	–	–	0.03	[69]
**Mouse Ago2-seed + 3′-sup**	0.2 × 10^8^		n.d.	n.d.		0.0005	–	–	0.01	[69]

### 3.2. Ago Modulates the Affinity of Binary Complexes for Target Strands

In a guide-target duplex, in absence of Ago all complementary bases contribute similarly to the affinity of the duplex. This is not the case if the guide strand is bound by Ago. Different parts of the guide-target duplex contribute to the overall binding affinity to various degrees [69]. It could be shown that the guide 5′-nucleotide is positioned in the Mid binding pocket and therefore not involved in base-pairing with the complementary target strand [7,13,38,40,69]. Wee *et al.* [69] additionally demonstrated that mismatches at guide position 8 and 9 or 10 and 11 have only very limited effects on the affinity of ternary complexes comprising fly Ago2 whereas mismatches in the seed at guide position 4 and 5 or in the 3′-supplementary region at position 15 and 16 are decreasing the affinity for a target strand significantly. A target associated with guide nucleotides 2–8 and 12–17 is bound as tightly as a fully complementary target strand. Wee *et al.* [69] proposed that miRNAs that do not have complementarity in the central region of the guide strand can evade the topological problem that has to be solved in case of siRNAs. The lack of contribution to binding affinity by the guide’s “tail” nucleotides 18–21 is consistent with structural data showing a block of base-pairing after 16 base-pairs by the N-terminal domain [6]. Experiments using hAgo2 emphasized the contribution of the seed region for the affinity of ternary complexes showing that base-pairing to guide nucleotides 2–8 is leading to highly stable ternary complexes while one additional base-pair with guide nucleotide 9 is decreasing binding affinity [39] (Table 4). It was suggested that base-pairing beyond the seed requires opening of the nucleic acid binding channel that could cause a lack of affinity. Schirle *et al.* [39] described this property of Ago as a kind of mechanistic switch to stabilize ternary complexes with seed-paired targets and differentiate between miRNA and siRNA target substrates. Supplementary base-pairing in the 3′-region of the guide exceeding guide nucleotide 9 is able to rescue binding affinity. These differential contributions of different guide-target duplex regions to the affinity of ternary complexes have important functional implications for Ago proteins. Without Ago weakening the interactions between the guide and the target strand, Ago would not be able to function as a multiple turnover enzyme: while a guide-target duplex in absence of Ago is highly stable, it does dissociate when bound to Ago to allow the association of the binary Ago-guide complex with a new target strand. In case of hAgo2, it could be demonstrated that the presence of target excess can even increase the rate of target release in a kind of strand invasion mechanism to enhance target turnover [46]. The destabilization of Ago-bound nucleic acids is probably important for most Ago proteins functioning at temperatures around 37 °C. Interestingly, in case of Ago proteins originating from thermophile prokaryotes the situation looks different. For example, *M. jannaschii* Ago-mediated cleavage is taking place at temperatures of above 75 °C whereas the melting point of the duplex used in the study is determined at 58 °C, indicating an Ago-induced stabilization of guide–target interactions [32].

**Table 4:** Binding affinities of different Ago-guide binary complexes for various target substrates.

Substrate	*K*_D_ (nM)	Reference
**hAgo2**		
21-Mer RNA	0.2	[46]
19-Mer RNA	204	[47]
20-Mer RNA	104	[47]
29-Mer RNA	43	[47]
Sod1-RNA (complementarity for guide nt 2–7)	20	[39]
Sod1-RNA (complementarity for guide nt 2–8)	1.9	[39]
Sod1-RNA (complementarity for guide nt 2–9)	4.0	[39]
Sod1-RNA (complementarity for guide nt 2–10)	2.4	[39]
**Fly Ago2**		
21-Mer RNA	0.004	[69]
**Mouse Ago2**		
21-Mer RNA	0.02	[69]

Transient binding experiments indicate that target release starts in the 3′-region of the guide and then proceeds to the seed region [46]. When cleavage products are accumulating, the probability rises that instead of binding to a new target, re-association of the 5′-portion of the cleavage product occurs. Since the cleavage product is base-paired to guide nucleotides 2–10 and the major portion of binding energy between binary complexes and target strand is provided by the seed region [69], the 5′-cleavage fragment is bound in a rather tight fashion [39]. Therefore, we propose a factor that is specifically increasing the dissociation of the target strand from the 5′-region of a guide strand but does not influence dissociation within the 3′-portion of the guide to prevent the removal of uncleaved target strands.

Although there is a high degree of similarity between different Ago proteins, there are important features that are different. These differences allow Ago proteins to fulfill a broad range of tasks in various organisms. Wee *et al.* [69] could show that Ago proteins dedicated to bind bulged miRNA targets are modulating the affinity of binary Ago-guide complexes for target strands in a way that is different from Ago proteins that are specialized in fully complementary targets that are subject to Ago-mediated cleavage (Table 3). Mouse Ago2 which is an Ago protein acting on miRNAs mostly dissociates from target strands before cleavage can take place. The dissociation rate constant does not change if mismatches in the 3′-region of the guide are introduced and they do not change the dissociation constants (Table 3). The situation looks completely different in case of fly Ago2 that is predominantly using siRNAs. Nearly every target that is bound is also cleaved and additionally mismatches introduced in the 3′-region are increasing the dissociation rate constant of ternary complexes by more than one order of magnitude (Table 3).

## 4. Conclusions

Since Ago proteins from all three domains of life share a remarkable structural conservation of the domains and their organization [40], mechanistic properties of Ago proteins are often analyzed with a particular Ago protein and applied to other Agos. Obviously, this is a legitimate strategy for major aspects of Ago-mediated silencing. However, the more structural and especially biochemical information is obtained from different Agos, it emerges that there are more differences than one initially thought. In-depth biochemical analyses of Ago proteins and their interaction partners can lead to insights that reveal how structurally highly homologous proteins can act in different pathways and differentiate between various substrates. Some Ago proteins strictly select their guide substrates by the 5′-nucleotide [43] whereas others show the same cleavage efficiencies using all four possible 5′-nucleotides [34]. The overall flexibility of different Ago proteins is comparable, however, there are significant differences concerning particular details [58]. Moreover, the modulation of the affinity between binary Ago-guide complexes and a matching target strand is dissimilar in Ago proteins acting in different pathways [69]. These findings show that more biochemical and structural research has to be done to pinpoint the origin of differences between Ago proteins. This information could furthermore be instrumental in the design of miRNA- and siRNA-based therapeutics. Several studies demonstrate that chemical modifications of therapeutically administered siRNAs or miRNAs lead to significantly improved serum stability and therefore to enhanced efficiency [70,71,72]. Biochemical insight into Ago protein binding to guide and target substrates, especially information gained from in-depth pre-steady state analyses, facilitate the prediction of the effect of siRNA modification on RISC function. Along these lines, *in vitro* assays used to examine pre-steady state and steady state kinetic parameters of Ago proteins enable a speedy and reliable system to compare such different chemical modifications and answer the question of how they might influence binding of guide and target substrates to Ago and subsequent Ago-mediated target cleavage. In other words, which modifications are tolerated by Ago? This would allow a precise prediction of the potency of potential therapeutic siRNA or miRNA candidates before testing in more costly tissue culture or animal systems. Besides, information gained from *in vitro* experiments with modified siRNAs and miRNAs might in the long run lead to the development of precise algorithms to predict efficient oligonucleotide therapeutics *in silico*.
